# Rewiring carbohydrate catabolism differentially affects survival of pancreatic cancer cell lines with diverse metabolic profiles

**DOI:** 10.18632/oncotarget.17172

**Published:** 2017-04-17

**Authors:** Tiziana Tataranni, Francesca Agriesti, Vitalba Ruggieri, Carmela Mazzoccoli, Vittorio Simeon, Ilaria Laurenzana, Rosella Scrima, Valerio Pazienza, Nazzareno Capitanio, Claudia Piccoli

**Affiliations:** ^1^ Laboratory of Pre-Clinical and Translational Research, IRCCS CROB, Referral Cancer Center of Basilicata, Rionero in Vulture, Italy; ^2^ Department of Clinical and Experimental Medicine, University of Foggia, Foggia, Italy; ^3^ Gastroenterology Unit, IRCCS “Casa Sollievo della Sofferenza”, Hospital San Giovanni Rotondo (FG), Foggia, Italy

**Keywords:** metabolic profiling, pancreatic cancer, mitochondrial metabolism, oxidative stress, metabolic therapy

## Abstract

An increasing body of evidence suggests that targeting cellular metabolism represents a promising effective approach to treat pancreatic cancer, overcome chemoresistance and ameliorate patient's prognosis and survival. In this study, following whole-genome expression analysis, we selected two pancreatic cancer cell lines, PANC-1 and BXPC-3, hallmarked by distinct metabolic profiles with specific concern to carbohydrate metabolism. Functional comparative analysis showed that BXPC-3 displayed a marked deficit of the mitochondrial respiratory and oxidative phosphorylation activity and a higher production of reactive oxygen species and a reduced NAD^+^/NADH ratio, indicating their bioenergetic reliance on glycolysis and a different redox homeostasis as compared to PANC-1. Both cell lines were challenged to rewire their metabolism by substituting glucose with galactose as carbon source, a condition inhibiting the glycolytic flux and fostering full oxidation of the sugar carbons. The obtained data strikingly show that the mitochondrial respiration-impaired-BXPC-3 cell line was unable to sustain the metabolic adaptation required by glucose deprivation/substitution, thereby resulting in a G2\M cell cycle shift, unbalance of the redox homeostasis, apoptosis induction. Conversely, the mitochondrial respiration-competent-PANC-1 cell line did not show clear evidence of cell sufferance. Our findings provide a strong rationale to candidate metabolism as a promising target for cancer therapy. Defining the metabolic features at time of pancreatic cancer diagnosis and likely of other tumors, appears to be crucial to predict the responsiveness to therapeutic approaches or coadjuvant interventions affecting metabolism.

## INTRODUCTION

Altered metabolism is a common property of invasive cancer cells that reprogram metabolic pathways to generate energy and macromolecules required for cellular growth [1]. Targeting cellular metabolism may represent, therefore, a selective strategy to improve the response to conventional drugs; moreover, the combination of chemotherapy with metabolic agents may overcome drug resistance in cancer therapy [2].

It is well known that tumor cells rely on glucose and glycolysis as a major source of energy instead of mitochondrial respiration, even in presence of oxygen [3]. This accounts for the increasing interest in fasting or dietary restriction to prevent or treat cancer [4] or even sugar free approaches to selectively kill cancer cells [5].

In this setting, pancreatic cancer could represent one of the best models to define a metabolic approach. The specific structure of this tumor confers a robust glycolytic activity to pancreatic cancer cells [6]. Pancreatic cancer displays one of the most extensive and poorly vascularized desmoplastic stromal reactions of all carcinomas, leading to tumor hypoxia and nutrient deprivation [7]. Pancreatic cancer requires new effective treatments due to its aggressiveness and chemoresistant properties [8].

A recent study already demonstrated that fasting potentiates gemcitabine effect in a pancreatic cancer xenograft model [9], consistent with another study showing that calorie restriction decreased murine and human pancreatic cell growth [10]. However, the latest findings in cancer metabolism are suggesting that not all cancer cells alter metabolic pathways in the same fashion [11]. Therefore, a preliminary metabolic characterization would be necessary to assess the effective sensitivity of cancer cells to metabolic targeting.

In this study, we selected two pancreatic cell lines hallmarked by a different metabolic profile as model-test to predict/verify their response to a potential therapeutic strategy affecting/rewiring energy metabolism.

## RESULTS

### Gene expression profile of PANC-1 and BXPC-3 cell lines

**Figure 1:**
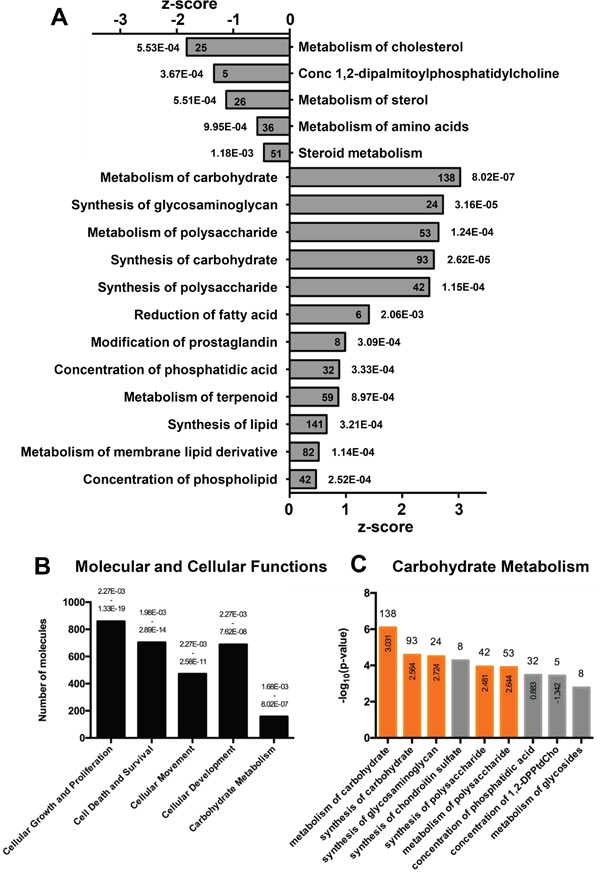
Ingenuity Pathway Analysis (IPA) of differentially expressed genes in PANC-1 and BXPC-3 cell lines **(A)** Predicted down-regulated and activated pathways in BXPC-3 cell line, compared to PANC-1. The x- and y-axes represent the z-score and the categories of pathways, respectively. *p*-value is shown aside of each bar; the number of molecules is reported inside the bars. **(B)** Top predicted biofunctions associated to genes up-regulated in BXPC-3 compared to PANC-1. The histogram represents the number of molecules involved in each annotated biofunction. *p*-value is shown on the top of each bar. **(C)** Top predicted biofunctions of carbohydrate metabolism represented in panel B. The histogram represents the -log_10_ (*p*-value) of each sub-annotation of carbohydrate metabolism. The number of molecules is reported on the top of each bar. Activation z-score, used by IPA to infer the activation state of functions, is reported inside the bar. Orange bars show positive activation of annotation.

We first performed a whole-genome expression profiling in two well-characterized pancreatic cancer cell lines, PANC-1 and BXPC-3, chosen among other cell lines for their similar growth conditions and because of their hallmarked geno/phenotype. The whole-genome expression analysis allowed the identification of 2280 genes differently modulated between the two cell lines (*P*< 0.001), 1204 up-regulated and 1076 down-regulated in BXPC-3 as compared with PANC-1 (Supplementary Table 1). This confirmed previous studies showing heterogeneity in gene expression among different pancreatic ductal adenocarcinomas or pancreatic cancer cell lines [12, 13]. Ingenuity pathway analysis (IPA) identified several cellular function categories linked to genes modulated in our dataset and, among the most relevant pathways, metabolism was highly represented (Figure 1A). Interestingly, carbohydrate metabolism was among the top five predicted functions with the highest number of genes involved, right after well known functions involved in cancer (Figure 1B) with several categories activated in BXPC-3 as compared to PANC-1 (Figure 1C). Moreover, *in silico* regulator effect analysis performed by IPA identified several molecules affecting glucose metabolism, under the control of AKT signaling (Supplementary Figure 1A). Western blotting for detection of total AKT and its Ser473-phosphorylated active form unveiled a significant higher expression level of AKT in BXPC-3 though the pAKT^Ser473^/AKT ratio was comparable in the two cell lines (Supplementary Figure 1B).

The identification of so many genes regulating metabolism and differentially expressed in PANC-1 and BXPC-3 let us to suppose a different metabolic machinery featuring these two cell lines. Therefore, we performed a systematic characterization of cellular metabolism of both PANC-1 and BXPC-3 also to validate the microarray analysis.

### Characterization of glycolytic and mitochondrial OxPhos activity in PANC-1 and BXPC-3

**Figure 2:**
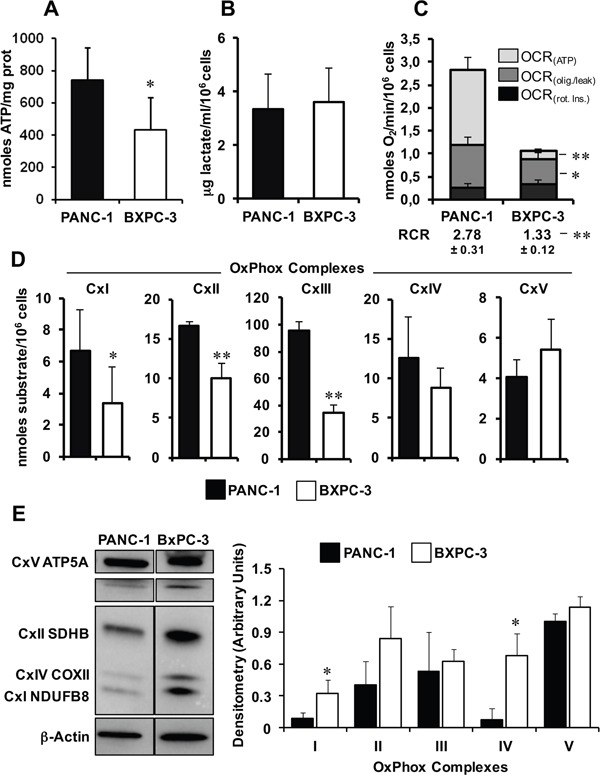
Comparative analysis of the metabolic profiles of PANC-1 and BXPC-3 cell lines **(A)** Cellular ATP content in PANC-1 and BXPC-3, measured as nmoles ATP/mg protein and expressed as means ± SEM of three independent experiments; *, P < 0.005. **(B)** Measurement of lactate release; cells were plated and, after 24 hours of incubation, the lactate concentration was determined in the medium as indicated in Material and Methods and normalized to the cell number. The reported data are means ± SEM of three independent experiments. **(C)** Graphical representation of oxygen consumption rate, expressed as nmoles O_2_/min/10^6^ cells and measured by high resolution respirometry as described in Materials and Methods. OCR_(ATP)_, respiratory activity obtained subtracting to the endogenous activity that attained after addition of the ATP-synthase inhibitor oligomycin; OCR_(olig./leak)_, respiratory activity in the presence of oligomycin corrected for the residual activity in the presence of the complex I inhibitor rotenone; OCR_(Rot.)_, residual activity in the presence of rotenone. The respiratory control ratio (RCR) was: (OCR_(ATP)_+OCR_(olig./leak)_)/OCR_(olig./leak)_. Values are reported as means ± SEM of at least four independent experiments under each conditions; *, P < 0.05; **, P < 0.001. **(D)** Measurement of the enzymatic activity of the OxPhos complexes. Cells were lysed and assayed spectrophotometrically as detailed in Materials and Methods. CxI, NADH dehydrogenase; CxII, Succinate dehydrogenase; CxIII, Coenzyme Q-cytochrome c oxidoreductase; CxIVcytochrome c oxidase; CxV FoF1-ATPase. The results are expressed as nmoles of substrate transformed/min/10^6^ cells and represent means ± SEM of three independent experiments for each conditions; *, P < 0.05; **, P < 0.01. **(E)** Expression of the OxPhos complexes. Left panel: representative immunoblot of the five OxPhos complexes (CI to CV) protein expression in PANC-1 and BXPC-3 cell lysates, using a cocktail of specific antibodies; β-actin was used as loading control. Histograms on the right: normalized densitometric analysis of the OxPhos complexes; values are means ± SEM from three independent experiments; * P < 0.01.

The bioenergetic competence/efficiency of PANC-1 and BXPC-3 cell lines was assessed measuring their intracellular steady-state ATP content that resulted significantly lower in BXPC-3 (Figure 2A). As the steady-state level of ATP depends on the equilibrium between the rates of ATP production and utilization, we next evaluated the two major cellular energy-generating pathways, i.e. glycolysis and mitochondrial respiration. While the production of lactate, as index of glycolysis, was similar between the two cancer cell lines (Figure 2B), high resolution oxymetry revealed that BXPC-3 cells displayed a significant lower rate of endogenous oxygen consumption rate (OCR) compared to PANC-1 (Figure 2C). This difference was still observed after correction for the residual oxygen consumption following addition of the complex I inhibitor rotenone and thus was largely attributable to the mitochondrial respiratory chain activity. Importantly, the difference between the resting OCR and that measured in the presence of the FoF1 ATP-synthase inhibitor oligomycin (OCR_olig/leak_ in Figure 2C), was also comparatively much lower in BXPC-3 indicating a remarkably low oxidative phosphorylation (OxPhos) efficiency therein (OCR_(ATP)_ in Figure 2C). This was further supported by the lower respiratory control ratio (RCR) obtained by the ratio of the overall rotenone-sensitive OCR (i.e. RCR_(ATP)_+OCR_(olig./leak)_ and the OCR_(olig./leak)_ in BXPC-3.

Next, we assayed the specific activity of each complex of the mitochondrial respiratory chain (complexes I to IV) and of the FoF1-ATP synthase (complex V) and confirmed in BXPC-3 a significant lower activity for complexes I, II and III as compared with those of PANC-1 (Figure 2D). No statistical significant difference was observed for complexes IV and V between the two cell lines. Surprisingly, assessment of the protein expression of the mitochondrial OxPhos complexes (CI to CV), using a cocktail of antibodies recognizing a specific subunit per each complex, resulted, counterintuitively, in a general higher protein expression in BXPC-3 compared to PANC-1, which was statistically significant for complexes I and IV (Figure 2E).

### Cellular redox state in PANC-1 and BXPC-3

**Figure 3:**
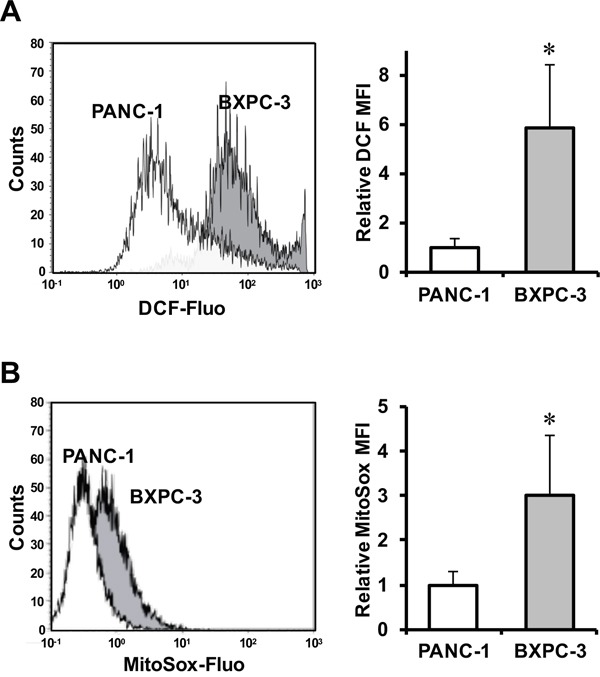
Evaluation of ROS production in PANC-1 and BXPC-3 cell lines Flow-cytometric analysis of ROS production assayed by the fluorescent peroxide probe DCF-DA **(A)** and the mitochondrial superoxide anion probe MitoSox **(B)** in PANC-1 (white areas) and BXPC-3 (grey areas) cells. Panels on left: representative flow-cytometric histogram plots. Bar histograms on the right: mean fluorescence intensity (MFI) normalized to that of PANC-1 of at least four independent biological replicates, expressed as means ± SEM; *, P < 0.01.

Mitochondrial dysfunction often results in alteration of reactive oxygen species (ROS) production [14]. For this reason, we compared the cellular redox homeostasis of PANC-1 and BXPC-3 at basal level, assessed by the peroxide fluorescent probe DCF, and found that BXPC-3 cells were characterized on an average basis by a six fold higher levels of intracellular ROS compared to PANC-1 (Figure 3A). The specific involvement of mitochondria in ROS production was assessed by the mitotropic O_2_^·-^-probe MitoSox, which displayed a significant three-fold higher fluorescence-related signal in BXPC-3 as compared with PANC-1 (Figure 3B).

### Mitochondrial membrane potential, mitochondrial mass and mtDNA copy number in PANC-1 and BXPC-3

**Figure 4:**
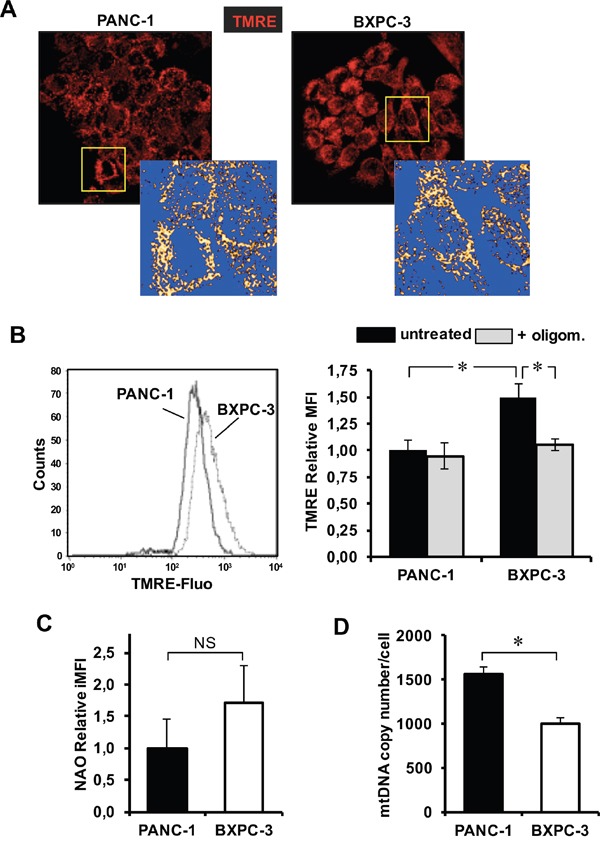
Evaluation of mitochondrial morpho-functional parameters, mitochondrial mass and mtDNA copy number in PANC-1 and BXPC-3 cell lines **(A)** Laser scanning confocal microscopy (LSCM) imaging of Δψ_m_ by the fluorescent probe TMRE. Digital magnification and rendering of the TMRE-related fluorescence (ImageJ software from http://imagej.nih.gov/ij/) of the mitochondrial compartment is also shown for the details indicated by the squares. The images are representative of at least three assays performed with independent cell preparations. **(B)** Flow-cytometric analysis of the Δψ_m_ PANC-1 and BXPC-3 stained with TMRE. Panel on left: representative flow-cytometric histogram plot. Bar histogram on the right: normalized MFI of at least four experiments, expressed as means ± SEM; the effect of pretreatment of oligomycin (2 μg/ml added 30 min before TMRE) is also shown; *, P < 0.05. **(C)** Flow-cytometric analysis of the mitochondrial mass assessed by the cardiolipin fluorescent probe NAO. The bar histogram shows the mean ± SEM of 3 independent experiments. **(D)** mtDNA copy number assessed by q-RT-PCR; the bar histogram shows values normalized to the nuclear DNA and are means ± SEM of 4 independent determination on biological replicates; *, P < 0.01. See Material and Methods for further details.

To implement the functional analysis of the mitochondrial compartment, we imaged, by confocal microscopy, the mitochondrial membrane potential (Δψ_m_) generated by the protonmotive activity of the electron transport chain, using the specific probe TMRE. At basal level the Δψ_m_-related TMRE fluorescence localized in dense and highly interconnected mitochondria and did not appear to differ in the two cell lines (Figure 4A). Pre-incubation of both PANC-1 and BXPC-3 cells with the uncoupler FCCP resulted repeatedly in large fading of the fluorescent signal (not shown). The Δψ_m_-related TMRE fluorescence, evaluated by flow-cytometry, resulted in a significant higher value in BXPC-3. Notably, oligomycin treatment caused a significant reduction of the Δψ_m_-related TMRE fluorescence in BXPC-3 while resulted ineffective in PANC-1 (Figure 4D). Next we assessed the mitochondrial mass using the fluorescent probe 10-N-nonyl acridine orange (NAO) which binds to the mitochondria-specific phospholipid cardiolipin. The result of the flow-cytometry analysis showed a higher NAO-related fluorescence signal in BXPC-3 though not reaching statistical significance as compared with PANC-1 (Figure 4C). To complement this observation, we further measured the relative mitochondrial DNA (mtDNA) content in the two cell lines by q-PCR. As shown in Figure 4D a significant 33 % lower mtDNA copy number/nuclear DNA was detected in BXPC-3 with respect to PANC-1.

### NAD metabolism in PANC-1 and BXPC-3

**Figure 5:**
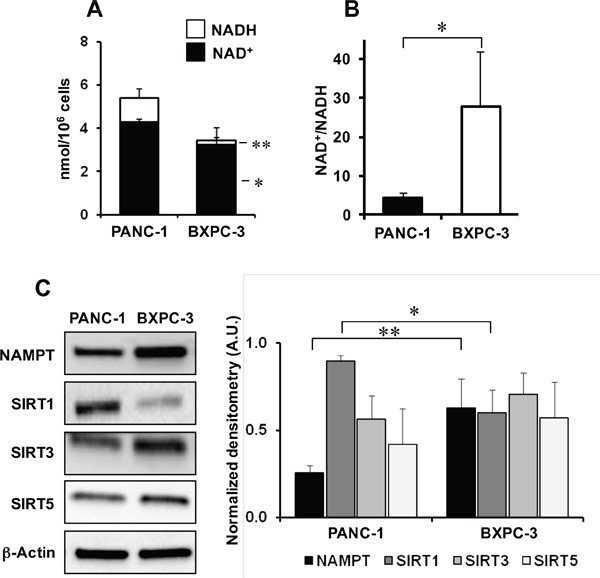
NAD metabolism in PANC-1 and BXPC-3 cell lines **(A)** Cellular content of NAD^+^ (black columns) and NADH (empty columns) normalized to the number of cells and expressed as mean ± SEM from three independent experiments; *, P < 0.05; **, P < 0.01. **(B)** NAD^+^/NADH ratio in PANC-1 and BXPC-3; *, P < 0.05 (n= 3 independent biological replicates). **(C)** Representative western blotting for protein expression levels of NAMPT, SIRT1, SIRT3, SIRT5 in PANC-1 and BXPC-3 and densitometric analysis performed for three independent experiments and expressed as means ± SEM; *, P < 0.05; **, P < 0.01.

As mitochondrial respiratory activity and redox balance interplay with the NAD^+^/NADH ratio, we analyzed aspects related to NAD metabolism. Figures 5A, 5B show that the total NAD content was significantly lower in BXPC-3 than in PANC-1 with a much higher NAD^+^/NADH ratio in the former.

Surprisingly, the protein expression of nicotinamide phosphoribosyltransferase (NAMPT), the rate limiting enzyme in the salvage pathway of the mammalian NAD synthesis, was higher in BXPC-3 as compared with PANC-1 confirming gene expression analysis (Figure 5C and Supplementary Table 1). As NAD is also the co-substrate in protein deacetylation processes catalized by sirtuins we compared the protein expression levels of the three major sirtuin isoforms, SIRT1, SIRT3 and SIRT5. The results in Figure 5C show that only a significant lower level of SIRT1 was observed in BXPC-3 as compared with PANC-1.

To verify the impact of the constitutive lower expression of SIRT1 combined with a lower content of NAD^+^ in BXPC-3 we inspected the acetylation state of the cell protein extract by western blotting with an antibody recognizing acetylated protein lysines. The result obtained clearly shows a different Ac-Lys profile between the two cancer cell lines with BXPC-3 exhibiting an overall higher protein acetylation as compared with PANC-1 (Supplementary Figure 2).

### Effect of glucose deprivation/substitution on pancreatic cancer cell lines

**Figure 6:**
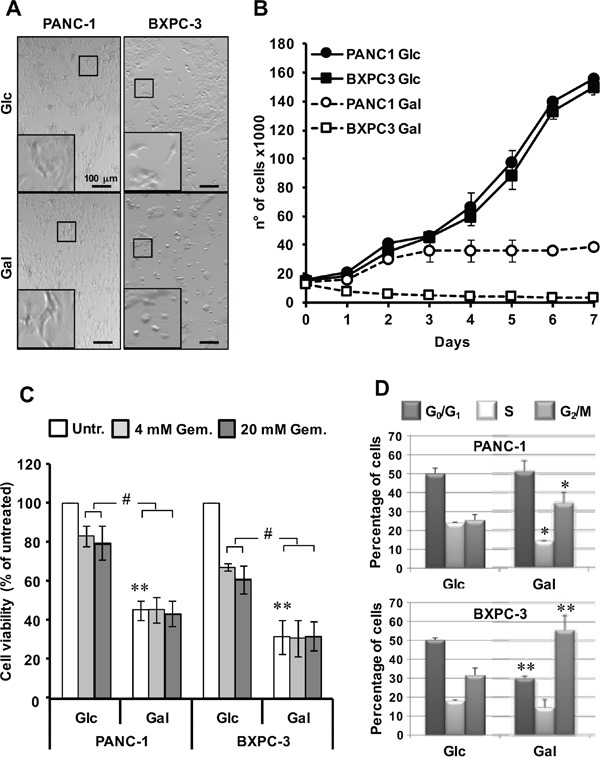
Effect of glucose deprivation/substitution on cell viability and cell cycle in PANC-1 and BXPC-3 cell lines **(A)** Morphological comparison of cultured cells in complete RPMI medium with 11.1 mM glucose (Glc) or with 11.1 mM galactose (Gal) substituting glucose for 24 h. The shown optical micro-photographs (magnification 50X) are representative of 3 independent biological replicates yielding similar results; digital magnifications of selected details are also shown. **(B)** Cell growth curves of cultured PANC-1 and BXPC-3; both cell lines were seeded at the same density in media with Glc or Gal (as in panel A) and counted every 24 h at the indicated times; the values shown are means of three independent ± SEM time-courses for each condition. **(C)** Effect of glucose deprivation/substitution on cell viability assessed by MTS assay, as described in Materials and Methods. Cell viability is expressed as the percentage (%) of untreated cells. The data shown are means ± SEM of at least 6 independent experiments; *, P < 0.05; ^#^, P < 0.01; ^##^, P < 0.005. **(D)** Cell cycle analysis: PANC-1 and BXPC-3 cultured with glucose or galactose were subjected to cell cycle analysis using the Muse Cell Analyzer. Histograms represent cell cycle distribution; *, P < 0.05; **, P < 0.01 for Glc vs Gal.

To test the metabolic resilience of PANC-1 and BXPC-3 cell lines, we evaluated the impact of glucose substitution with galactose on cell viability. Differently from glucose, the slower entry of galactose in the Embden-Meyerhof pathway forces cells to oxidize pyruvate to maximize the utilization of the available carbon source [15]. Consequently, aerobic glycolysis is strongly depressed and ATP production is almost completely reliant on mitochondrial oxidative phosphorylation. As shown in Figure 6A, a qualitative microscopic observation indicated that withdrawal/substitution of glucose with galactose reduced the cell number and altered morphology in BXPC-3 whilst no apparent alteration in PANC-1 was evident.

A cell growth assay confirmed a selective marked sensibility of BXPC-3 to glucose withdrawal/substitution. (Figure 6B). The results obtained show that as compared with the growth in glucose (where both PANC-1 and BXPC-3 displayed a similar growth kinetics), substitution of glucose with galactose resulted for PANC-1 after 3 days in a block of growth which persisted up to 7 days. Conversely, the growth of BXPC-3 in galactose was strongly inhibited since the first day of culture with the cell number declining progressively onwards. Since the growth of pancreatic cancer cells relies on the availability of glutamine [16], we tested also the effect of glutamine withdrawal on the growth of both cell lines. As shown in Supplementary Figure 3 both PANC-1 and BXPC-3 displayed a similar block of growth starting from the 2^nd^-3^rd^ day of glutamine deprivation persisting up to the 7^th^ day.

Unlike the growth assay, the MTS assay indicated a loss of viability in both cell lines with BXPC-3 resulting slightly but significantly more sensitive to glucose deprivation/substitution than PANC-1 (Figure 6C). Notably, the inhibitory effect of glucose substitution with galactose on cell viability was even greater than that elicited by the conventional chemotherapeutic agent gemcitabine tested at two different concentrations (4 and 20 μM). Treatment with gemcitabine of both cell lines growing in galactose did not result in additive effect on cell viability (Figure 6C).

Cell cycle analysis of cells grown in galactose substituting glucose displayed a shift in the G2/M checkpoint in both cell lines that was much more pronounced in BXPC-3 (Figure 6D). Moreover, a significant reduction of the S and G0/G1 phases was observed for PANC-1 and BXPC-3 respectively. Conversely, gemcitabine exerted only a modest shift of PANC-1 in the G0/G1 phase (not shown).

**Figure 7:**
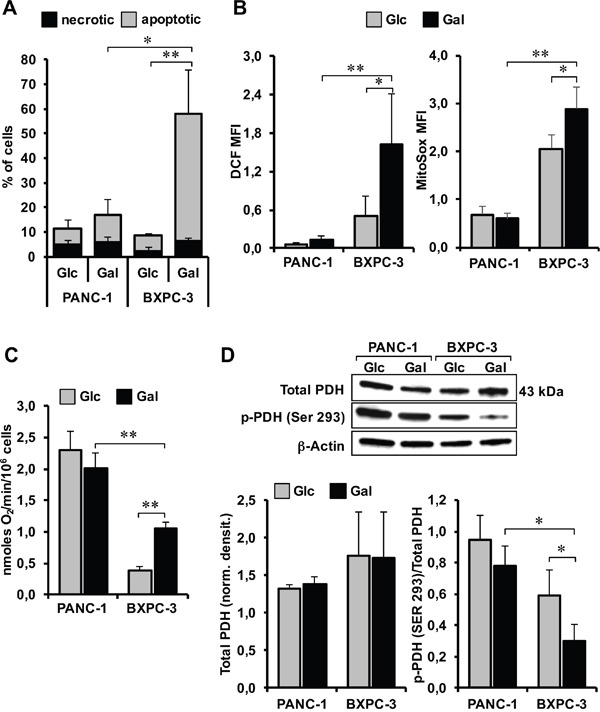
Effect of glucose deprivation/substitution on apoptosis, ROS generation, oxygen consumption and activation state of PDH in PANC-1 and BXPC-3 cell lines **(A)** Measurement of apoptotic cells performed by flow-cytometric analysis in both cell lines cultured with Glc or Gal as in Figure 6. Data are the mean ± SEM of three independent experiments and are expressed as percentage (%) of total analyzed events (*p<0.05 *versus* PANC-1; **p<0.01 *versus* BxPC-3). **(B)** Flow-cytometric analysis of ROS production assayed by the fluorescent probes DCF and MitoSox in PANC-1 and BXPC-3 cells cultured in either glucose (Glc) or galactose (Gal) containing medium (representative of three independent experiments under each condition). The bar histograms show means ± SEM of the probe-related MFI of three independent experiments under each condition; *, P < 0.05; **, P < 0.01. **(C)** Effect of glucose deprivation/substitution on the endogenous oxygen consumption rate (i.e. OCR_(ATP)_+OCR_(olig./leak)_ as in Figure 2C). The values reported are means ± SEM of at least four independent experiments; **, P < 0.01. **(D)** Western blot analysis of total PDH E1 and p-PDH E1 (Ser293) on whole protein cell extract. Top panel: representative blots. Bottom bar histograms: densitometric analysis showing mean values ± SEM of total PDH normalized to β-ACTIN (left histogram) and of the p-PDH/PDH ratio (right histogram) of 3 independent experiments; *, P < 0.05. See legend to Figure 6 and Materials and Methods for further details.

To deepen the nature of the observed loss of the cell viability, we performed an apoptotic assay under condition of glucose deprivation/substitution. The results shown in Figure 7A indicate that glucose deprivation/substitution caused a large induction of apoptosis specifically in BXPC-3 with only a marginal effect on PANC-1. This differential effect is likely linked to a further enhanced oxidative stress caused by glucose deprivation/substitution selectively in BXPC-3 as shown in Figure 7B for both peroxide and mitochondrial superoxide production. Interestingly, glucose deprivation/substitution caused a significant increase of mitochondrial respiration in BXPC-3 whereas that of PANC-1 was unchanged (Figure 7C).

To verify whether galactose-conditioning primed cells to be more prone to oxidize pyruvate we performed western blotting analysis of pyruvate dehydrogenase and of its inactive E1 Ser293 phosphorylated state. As shown in Figure 7D while the expression level of PDH was comparable in the two pancreatic cancer cell lines, substitution of glucose with galactose caused a decrease in the phosphorylation state of PDH, which was significantly larger in BXPC-3 as compared with PANC-1.

The content of ATP was not significantly affected by glucose deprivation/substitution, though showing a trend to decrease in both cell lines (data not shown). No significant change was observed in mitochondrial mass assessed by NAO neither in the mtDNA copy number under galactose-conditioning (data not shown). To note, glucose deprivation/substitution regimen caused a large significant increase of the pAKT/AKT ratio in PANC-1 but was ineffective in BXPC-3 (Supplementary Figure 1B).

## DISCUSSION

Although it is generally assumed that cancer cells display a “distinctive” metabolism [5], recent studies are revisiting the “Warburg effect dogma”, demonstrating that not all cancer cells share a similar metabolic profile even within the same cancer phenotype [17]. Therefore, a systematic metabolic characterization is emerging as desirable when a tumor is diagnosed, in order to expand the therapeutic strategy, wherever possible.

In this study, we performed a biochemical characterization of two pancreatic cancer cell lines, PANC-1 and BXPC-3, whose genotype and phenotype have been previously described, characterized by a peculiar degree of adhesion, migration and invasion ability, tumorigenic and angiogenic potential [18].

Both proteomic and metabolomic profiling had already demonstrated a strong involvement of metabolic pathways in pancreatic cancer cell lines, suggesting the possibility to predict sensitivity of tumors to metabolic inhibitors [19, 20]. Our gene profile analysis confirmed that a number of genes, differentially expressed between the two cell lines, are linked to metabolic pathways. The significant difference in ATP levels between BXPC-3 and PANC-1 suggested a different ATP-production activity. In order to make a functional validation of these data, we tested glycolytic activity of both cell lines. Although no difference was observed in the amount of lactate released by the two cell lines, analysis of the mitochondrial respiratory activity revealed, instead, a significant difference. In particular, BXPC-3 displayed a much lower oxygen consumption rate together with a lower activity of the respiratory chain complexes. However, the protein expression level of the respiratory chain complexes was higher in BXPC-3 as compared to PANC-1 in spite of significant lower mtDNA copy number.

These apparently conflicting results might be due to a compensatory over-expression of the respiratory chain complexes in BXPC-3 not resulting in a functional gain of the mitochondrial OxPhos activity possibly caused by mutations of the mitochondrial DNA (coding for catalytic subunits of complexes I, III and IV). This event often occurring in tumorigenesis [21] has been recently demonstrated by an *extensive* mtDNA sequencing analysis on a unique cohort of patient-derived pancreatic cancer cell lines and combined with metabolomic and phenotypic characterization [22]. In this study, 24 somatic mutations in the mtDNA of 12 patient-derived pancreatic cancer cell lines were identified. A further 18 mutations were identified in a targeted study of ~1000 nuclear genes important for mitochondrial function and metabolism. Comparison with reference datasets indicated a strong selection bias for non-synonymous mutants with predicted functional effects. Phenotypic analysis showed metabolic changes consistent with mitochondrial dysfunction, including reduced oxygen consumption. Most notably, the major number of mutations occurred in mtDNA genes coding for subunits of CxI.

To notice, the observed higher expression of the mtDNA-encoded subunit COXII of the cytochrome c oxidase (CxIV) in BXPC-3 would imply a higher transcriptional activity of the mitochondrial genome. Consistent with this hypothesis is the reported observation of a significant difference in the redox homeostasis between the two cell lines, with BXPC-3 producing higher ROS levels, compared to PANC-1, both as cellular peroxide and most notably as mitochondrial superoxide anion. Enhanced ROS production is, indeed, a hallmark of dysfunctions in the respiratory chain [23] and a trigger of mitochondrial biogenesis [24]. All these alterations did not affect the mitochondrial network morphology, which was comparable between the two cancer cell lines, albeit some increase of the mitochondrial mass was observed in BXPC-3.

Notably, the extent of the Δψ_m_ was higher in the low-respiring BXPC-3 cells as compared to PANC-1. However, it has to be considered that the Δψ_m_ in addition to be generated by the protonmotive activity of the respiratory chain can also be generated by the reverse ATP-hydrolase activity of the H^+^-FoF1-ATP-synthase [25]. It is, therefore, possible that in BXPC-3 cells part of the glycolytic ATP is used to maintain a high Δψ_m_. To support this possibility is the observation that in the presence of oligomycin, blocking both the ATP-synthase and ATP-hydrolase of complex V, the resting Δψ_m_ is decreased specifically in BXPC-3.

To complete this preliminary characterization, we also measured the intracellular total NAD and its redox state. The results obtained show that in BXPC-3 cells the NAD^+^/NADH ratio is much higher than in PANC-1 setting in the former a 20 mV more positive intracellular redox potential. This indicates a distinctive pro-oxidative condition in BXPC-3 cells as supported by the observed higher steady-state production of ROS therein. Most notably, the total content of cellular NAD was significantly lower in BXPC-3 further indicating a defective OxPhos activity also caused by a limited availability of the main redox cofactor. In addition to serve as a redox coenzyme, NAD^+^ is also a co-substrate in a number of enzymatic reactions among which protein deacetylation and poly(ADP-ribose) polymerization are the best characterized [26]. These two reactions, catalyzed by members of the sirtuins family and by PARP enzymes, contribute to degrade NAD. Thus the stationary level of NAD results from the balance between its degradation and biosynthesis. It has been shown that pancreatic cancer cells mainly rely on the savage pathway of NAD synthesis where NAMPT is the rate committing enzyme [27] found to be overexpressed in many cancers and to correlate with their development [28]. In this study, we found in BXPC-3 a significant higher expression of NAMPT at the transcript and protein level as compared with PANC-1 cells. Among the members of the sirtuin family, SIRT1, the most abundant sirtuin in the cell, resulted down regulated in BXPC-3. This is consistent with the negative effect exerted by an altered redox status, as that found in BXPC-3 respect to PANC-1, on SIRT1 level and activity through its post-translational modification [29]. The relative lower level of SIRT-1 and NAD^+^ found in BXPC-3 translated in an altered protein acetylation profile. This point warrens further investigation given the emerging role played by NAD-utilizing enzymes in maintaining redox homeostasis, in controlling metabolism and in the development and progression of cancer [30].

Taken together, all these data demonstrated that pancreas carcinoma-derived cell lines display different metabolic profiles with cell lines like BXPC-3 hallmarked by major alterations in the mitochondrial oxidative functions. The following goal of our study was, therefore, to find/suggest a strategy to hit cancer cells, taking advantage of all these features. Interestingly, a deeper analysis of gene expression profile highlighted the regulation of carbohydrates metabolism as one of the top biofunctions featuring BXPC-3 compared to PANC-1, identifying several genes involved in glucose metabolism, most of them under control of AKT. Consistently, we found a significant higher expression of AKT in BXPC-3 though the P-AKT/AKT ratio was similar in both cell lines. AKT is known to promote glycolysis, enhancing transcription of glycolytic enzymes and cell survival [31]. Then, we better investigated the effect of glucose deprivation to cell survival in both cell lines. It is known that resistance to gemcitabine, the standard therapy for treatment of patients with pancreatic cancer [32], represents one cause of therapeutic fail in pancreatic cancer treatment. To date, caloric restriction has been proposed as adjuvant able to improve cancer therapeutics [33]. However, a regime of caloric restriction, even if carefully controlled, may not encounter full compliance, for obvious reasons, and even not to be beneficial in seriously debilitated patients. An alternative dietary approach might be to rewire cell metabolism providing alternative carbohydrates in place of glucose. The entry of galactose in the glycolytic flux, requiring four additional slow enzymatic steps, forces cells to compensate a slower catabolic flux by increasing efficiency in terms of ATP production. Therefore, galactose-derived pyruvate is not converted in lactose but is fully oxidized within mitochondria thereby driving OxPhos. Substitution of glucose with galactose in the media of cultured cells was found to enhance mitochondria biogenesis and to change their networked morphology [15]. However, if cells have a defective OxPhos system or have adopted prominently an aerobic glycolytic profile pushing them toward an oxidative metabolism might result in growth impairment or even cell death as a consequence of the energy crisis. Given these premises, as proof of principle, we tested the effects of glucose deprivation/substitution on the cell growth of the two pancreatic cancer cell lines. The different metabolic profile of PANC-1 and BXPC-3 resulted in a marked differential sensitivity to glucose deprivation/substitution that was evident as early as after 24 h of glucose deprivation/substitution in BXPC-3 that also displayed major morphological changes and a much stronger apoptosis priming. This is likely linked, under this condition, to an overproduction of ROS in BXPC-3, which in turn might correlate to the observed enhanced oxygen consumption rate fostered by an enhanced pyruvate oxidation as inferable from the observed activation of the pyruvate dehydrogenase.

Interestingly, prolonging the period of glucose deprivation/substitution resulted in a progressive decline of the BXPC-3 cell growth whereas that of PANC-1 displayed a block after 48-72 h that persisted till 7 days of treatment. This result suggests that galactose conditioning might have a later impact also on the cell physiology of PANC-1 though resulting in a different outcome as compared with BXPC-3. Consistent with this conclusion is the observation that glucose deprivation/substitution had a significant impact on the cell cycle of both cell line albeit with some subtle differences.

There is a growing appreciation that metabolic signals are integrated and coupled to cell cycle progression. However, the molecular wiring that connects nutrient availability, biosynthetic intermediates and energetic balance to the core cell cycle machinery remains incompletely understood [34]. The notion that is emerging is that while the G0/G1/S phases rely on a balanced distribution of glycolysis and OxPhos, the G2/M phases are more closely dependent on OxPhos. Moreover, the cell cycle check-points are sensitive to the cellular redox tone [35]. In particular, a pro-oxidative state appears to cause a G2/M block preluding to arrest of proliferation and apoptotic commitment. Our interpretation, is that the deregulation of the cell cycle observed in both the pancreatic cancer cell lines with galactose substituting glucose may result from the combination of a change in the nutrient availability and an enhanced production of ROS.

To notice, when cell viability was tested by the common MTS assay both cell lines were similarly sensitive to glucose deprivation/substitution, which resulted even more efficient than treatment to conventional chemotherapeutic agent. In particular, glucose deprivation/substitution alone, independently of co-treatment with gemcitabine at different concentrations, was able to drastically affect cell survival in both cell lines. This result obtained by the MTS assay would suggest additional more general cytotoxic effects of glucose deprivation/substitution irrespective of the specific cell metabolic profile. However, it has to be taken into account that the MTS assay (and similar colorimetric assays) are prone to a number of variables that may lead to over/under estimation of the real cell viability. Among these are the level of intracellular NADH and the activity of the succinate dehydrogenase (complex II) [36]. Thus, in keeping these caveats, we would de-emphasize the results obtained with the MTS assay and give more relevance to the finding that cancer cells with a compromised mitochondrial respiratory activity are strikingly more sensitive to be negatively affected by interventions diverting their metabolism far from glycolysis.

**Figure 8:**
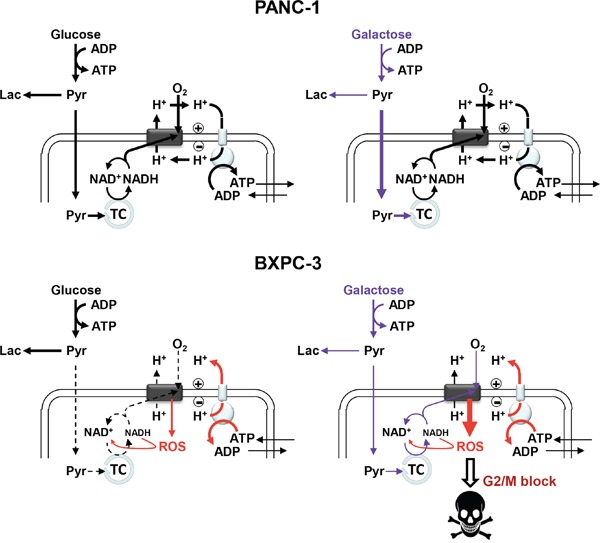
Schematic overview of the main metabolic functions and their alterations following glucose substitution with galactose in PANC-1 and BXPC-3 The schemes highlight the features of the glycolytic and OxPhos pathways with the thickness of the arrows proportional to the underlying flux. (Pyr: Pyruvate; Lac: Lactate; TC: tricarboxylic acid cycle; ROS: Reactive Oxygen Species). See Discussion for further details and explanation.

The results so far discussed confirm the potential cytotoxic effect ensued by manipulation of the catabolic carbon source on culture cancer cell lines. A schematic synthesis of the metabolic functions and their alterations in PANC-1 and BXPC-3 cell lines comparatively reported in this study is shown in Figure 8. It must be highlighted that in this study the cells were not subjected to conditions mimicking caloric restriction since glucose was substituted with equimolar galactose. Moreover, glucose deprivation/substitution translates specifically in BXPC-3 in cell cycle G2/M shift and no activation of AKT.

Our results show that the metabolic profile affects the drug responsiveness in pancreatic cancer cell lines, as also previously demonstrated by our group in other chemoresistant tumor cells [37]. Therefore, a preliminary characterization of the energy production efficiency and metabolic sources in cancer cells or tissues at time of diagnosis, could be relevant in order to design, if possible, new alternative/coadjuvant therapeutic strategies affecting metabolism.

## MATERIALS AND METHODS

### Cell culture

PANC-1 and BXPC-3 cells were purchased from American Type Culture Collection (ATCC, Manassas, VA, Usa) and cultured at 37 °C in a 5% CO_2_ humidified atmosphere in complete RPMI medium containing 11.1 mM glucose or in glucose deprived medium (GIBCO®) where 11.1 mM galactose was added; both media were supplemented with 10% fetal bovine serum, penicillin–streptomycin (100 U/ml), 2 mM glutamine. Where indicated, RPMI medium (with glucose) without glutamine was used. Gemcitabine and galactose were purchased from Sigma Aldrich (St. Louis, MO, USA) and added to medium for the indicated doses and time periods. The morphology of cells was observed at inverted optical microscope (Axio Vert A1, Zeiss).

### Gene expression profile

Total RNA was extracted with RNA easy Mini kit (Qiagen, Hilden, Germany), according to the manufacturer's instruction, the concentration determined on a Nanodrop spectrophotometer (Nano-Drop, Wilmington, DE), and quality assessed with the Agilent RNA 6000 Nano Kit on an Agilent 2100 Bioanalyzer (Agilent Technologies, Milan, Italy). For each sample, 300 ng of total RNA was reverse transcribed to synthesize cDNA and biotinylated cRNA according to the Illumina TotalPrep RNA amplification protocol (Ambion; category n. IL1791). Hybridization of 750 ng of cRNA on Illumina HumanHT12 v4.0 Expression BeadChip array (Illumina Inc.), staining and scanning were performed according to the standard protocol (Illumina Inc.). BeadChip was dried and scanned with an Illumina HiScanSQ system (Illumina Inc.). The intensity files were loaded into the Illumina Genome Studio software for quality control and gene expression analysis. Quantile normalization algorithm was applied on the data set to correct systematic errors: values below a detection score of 0.05 were filtered out and missing values were imputed. Microarray data (raw and normalized) were submitted to Array Express (accession number E-MTAB-4540). PANC-1 cell lines were selected as “control” cell line and compared with BXPC-3. Differently expressed genes (DEGs) were selected with differential score (DiffScore) cutoff set at ±13 (p<0.05) and logFC of +/- 0.5. The DEGs list included 2280 genes (1204 up-regulated and 1076 down-regulated), and was used to evaluate the functional behavior in terms of Biological Processes performing an enrichment analysis with Ingenuity Pathway Analysis (IPA) - (Ingenuity Systems, Redwood City, CA; http://www.ingenuity.com).

### Mitochondrial DNA quantification

The measurement of mtDNA copy number, relative to nuclear DNA copy number was determined amplifying mitochondrial tRNA-Leu(UUR) and nuclear-encoded β2m (beta-2-microglobulin) genes. Total DNA was extracted with QIAmp DNA mini kit (Qiagen, Hilden, Germany), according to the manufacturer's instructions and quantified by Nanodrop® ND-1000 spectrophotometer. Realtime PCR reactions were performed on a LightCycler^®^ 480 real-time PCR Instrument (Roche Diagnostic). 2 μl of total DNA (3 ng/μl) were added to Light Cycler^®^ 480SYBR Green I Master (Roche Diagnostics) and primers pair in a total volume of 25 μl. Human β2m forward (5′-TGCTGTCTCCATGTTTGATGTATC T-3′) and β2m reverse (5′-TCTCTGCTCCCC ACCTCTAAGT -3′) or tRNA-Leu(UUR) forward (5′-CACCCAAGAACAGGGTTTGT—3’) and tRNA-Leu(UUR) reverse (5’-TGGCCATGGGTATGTTGTTA-3’) primers were used. The following protocol was used: 50°C for 2 minutes, 95°C for 10 minutes, 40 cycles of 95°C for 15 seconds and 62° for 60 seconds. A dissociation curve was also calculated for each sample to ensure presence of a single PCR product. To determine the mitochondrial DNA content, relative to nuclear DNA the following equations were used: ΔCT=(nucDNA CT – mtDNA CT); relative mitochondrial DNA content = 2 × 2^ΔCT^ [38].

### Measurement of total cellular ATP

PANC-1 and BXPC-3 were collected by trypsinization and centrifugation. Cellular ATP was then extracted by adding boiling water to cellular pellet. After vortexing and centrifugation (12,000 g for 5 min at 4°C), 20 μl of the supernatant was added to 100 μl of luciferin/luciferase (rL/L) reagent. ATP content was determined using the ENLITEN® ATP Assay system Bioluminescence detection Kit (Promega), according to the manufacterer's instructions on a Victor2030 Explorer (PerkinElmer) and normalized on protein content.

### Lactate measurements

A Lactate Colorimetric Assay Kit (Abcam, Cambridge, MA, USA) was used following the manufacturer's protocol and the lactate concentration detected was normalized to cell number.

### Measurement of endogenous respiration rates in intact cells

Cultured cells were detached by trypsinization, washed in PBS, harvested by centrifugation, and immediately assessed for O_2_ consumption by a Clark-type electrode (Hansatech) in a thermostated gas-tight chamber equipped with a stirring device. Cells were assayed in 50 mM KPi, 10 mM Hepes, 1 mM EDTA (pH 7.4) at 37°C; after attainment of a stationary endogenous substrate-sustained respiratory rate, 1 μg/ml of oligomycin and 400 nM carbonylcyanide-p-trifluoromethoxyphenylhydrazone (FCCP) were added sequentially within a 10-minute interval. The rates of O_2_ consumption (OCRs) were corrected for 1μM rotenone-insensitive respiration. The OCRs were normalized to the cell number. Oligomycin, FCCP and Rotenone were purchased by Sigma Aldrich (St. Louis, MO, USA).

### Mitochondrial respiratory complexes activity

The specific activities of each complex of the respiratory chain (CI-CV) were assayed spectrophotometrically on frozen–thawed and ultrasound-treated cells as previously described [39; 40].

### Flow cytometric detection of mitochondrial membrane potential, mitochondrial mass and ROS

To measure mitochondrial membrane potential (ΔΨ_m_) and mitochondrial mass, we used 1 μM tetramethylrhodamine ethyl ester (TMRE) and 50 nM acridine orange 10-nonyl bromide (NAO), respectively. Intracellular ROS were detected using 4 μM 2,7-dichlorofluorescin diacetate (DCFH-DA); superoxide, specifically produced by mitochondria, was detected by 5 μM MitoSOX™. All the probes, purchased from Molecular Probes (Eugene, OR, USA) were added to cell suspension and incubated, protected from light, at 37°C for 15 min. The flow cytometric analysis was performed by FACSCalibur™ flow cytometer, (Becton Dickinson, BD) or Navios (Beckman Coulter). The emitted fluorescent signal of 10,000 events for each sample was acquired and analyzed with the CellQuest software or Kaluza Analysis 1.3.

### Live cell imaging of mtΔΨ

Cells cultured at low density on fibronectin-coated 35-mm glass-bottom dishes were incubated for 20 minutes at 37°C with 2 μM TMRE (Molecular Probes, Eugene, OR) to monitor mtΔΨ. Stained cells were washed with PBS and examined by a Nikon TE 2000 microscope (images collected using a 60X objective [1.4 NA]) coupled to a Radiance 2100 dual-laser (4-line Argon-Krypton, single-line Helium-Neon) confocal laser scanning microscopy system (Biorad). Acquisition, storage, and analysis of data were performed with LaserSharp and LaserPix software from Biorad or ImageJ1.48u (Wayne Rasband, NIH, USA, http://imagej.nih.gov/ij).

### Measurement of NAD^+^ and NADH

Cells, collected by trypsinization and centrifugation, were suspended in the extraction buffer (20mM NaHCO_3_, 100mM Na_2_HCO_3_, 10mM Nicotinamide, 0.05% Triton X-100, pH 10.3), frozen and thawed. After centrifugation, the supernatants were collected and assayed immediately. NAD or NADH concentrations were measured by a cyclic enzyme reaction system in which alcohol dehydrogenase reduces dichlorophenol indolphenol (DCIPIP) through the intermediation of phenazine methosulfate (PMS). The reaction mixture consisted of 0.63 ml of 100 mM phosphate Buffer (pH 7.5), 0.03 ml of 30 mM phenazine methosulphate (PMS), 0.04 ml of 0.6 mM dichlorophenol indolphenol (DCIPIP), 0.1 ml of 95% ethanol, 5 units ADH. The reaction was started by the addition of the sample. Reduction of the blue-colored DCPIP to colorless DCPIPH_2_ was measured by recording the decrease in absorbance at 600 nm. The concentration of NADH and NAD^+^ in each extract was determined by comparing sample values to standard curves generated from samples containing known amounts of NADH and NAD^+^ that had been cycled under identical conditions as the samples.

### Western blotting analysis

Aliquots containing 40 μg of proteins from each lysate cells were subjected to SDS polyacrylamide gel electrophoresis and transferred to a polyvinylidene difluoride membrane (Bio-Rad Laboratories; Hercules, CA, USA) using Trans Blot Turbo Transfer System. Membranes were probed with the following primary antibodies: MitoProfile Total OXPHOS Human WB Antibody cocktail, Pyruvate Dehydrogenase E1-alpha (PDH) and pPDHSer^293^ (1:500; Abcam Cambridge, UK), Acetylated Lysine (1:1000 Novus, UK), pAKTSer^473^, AKT, NAMPT, SIRT1, SIRT3, SIRT5 (1:1000; Cell Signaling Technology) and β-ACTIN (1:5000; SIGMA Aldrich, St. Louis, MO, USA). After incubation with corresponding suited horseradish peroxidase-conjugated secondary antibody (1:2500; Cell Signaling Technology) signals were developed using the enhanced chemiluminescence kit (ClarityTM Western ECL Substrate, Bio-Rad) and the ChemiDoc Imaging System XRS + (BioRad) and analysed with the Image Lab 4.1 software. The intensity of bands corresponding to Mitoprofile, Acetylated Lysine, NAMPT, SIRT1, SIRT3 and SIRT5 was normalized to the β-ACTIN signal while phosphorylated AKT and PDH was normalized to total protein.

### Cell viability assays

Cell growth curves of PANC-1 and BXPC-3 were performed as previously described [41]. For MTS assay, cells were seeded in a 96-well culture plate in control medium and then cultured in glucose or galactose ± gemcitabine for 24h at the indicated doses. Cell viability was measured using solutions of a novel tetrazolium compound (3-(4,5-dimethylthiazol-2-yl)-5-(3-carboxymethoxyphenyl)-2-(4-sulfophenyl)-2H-tetrazolium, inner salt (CellTiter 96® AQueous MTS Reagent Powder, Promega) and the electron coupling reagent, phenazine methosulfate, PMS (Sigma Aldrich, Saint Louis, MO, USA). MTS is bioreduced by cells into a formazan product that is soluble in tissue culture medium. The absorbance of the formazan at 490 nm was measured directly from 96-well assay plates using the Plate Reader (das srl, Italy) with a reference filter at 630 nm. All measurements were performed in triplicate for each assay.

### Apoptosis assay

Apoptosis was detected by flow cytometry (FACSCalibur™, BD) following staining of cells for Annexin-V-FITC and PI (BD Pharmingen), after 24h of glucose deprived medium cultured. Three independent experiments were carried out. 10,000 events were collected per sample. Annexin V and PI negative cells were considered viable; both cells in early (Annexin V positive, PI negative) and late (Annexin V positive, PI positive) apoptosis were assumed as apoptotic; Annexin V negative, PI positive cells were considered necrotic.

### Cell cycle assay

Cells were harvested at least 3 hours before the experiments. After fixation with 1ml of 70% cold ethanol at -20°C, as indicated by the Muse Cell Cycle Kit User's Guide, 200μl of ethanol-fixed cells were incubated with PI and RNAse A for 30 minutes at room temperature, before loading on Muse Cell Analyzer (Millipore, Italy) according to the supplied staining protocol.

### Statistical analysis

Experimental data are expressed as the mean ± standard error mean (SEM) or mean± standard deviation (SD). Data were compared by the unpaired Student's t-test or one-way Anova, followed by Bonferroni test; a *p* value < 0.05 was accepted as statistically significant.

## SUPPLEMENTARY MATERIALS FIGURES AND TABLES




