# Supplementary material for: Rewiring carbohydrate catabolism differentially affects survival of pancreatic cancer cell lines with diverse metabolic profiles
Source: Oncotarget. 2017 Apr 17;8(25):41265–81. doi: 10.18632/oncotarget.17172 (PMC5522241; doi:10.18632/oncotarget.17172)
Supplement: Supplementary file 1 [file oncotarget-08-41265-s001.pdf]

Rewiring carbohydrate catabolism differentially affects survival of pancreatic cancer cell lines with diverse metabolic profiles

SUPPLEMENTARY MATERIALS

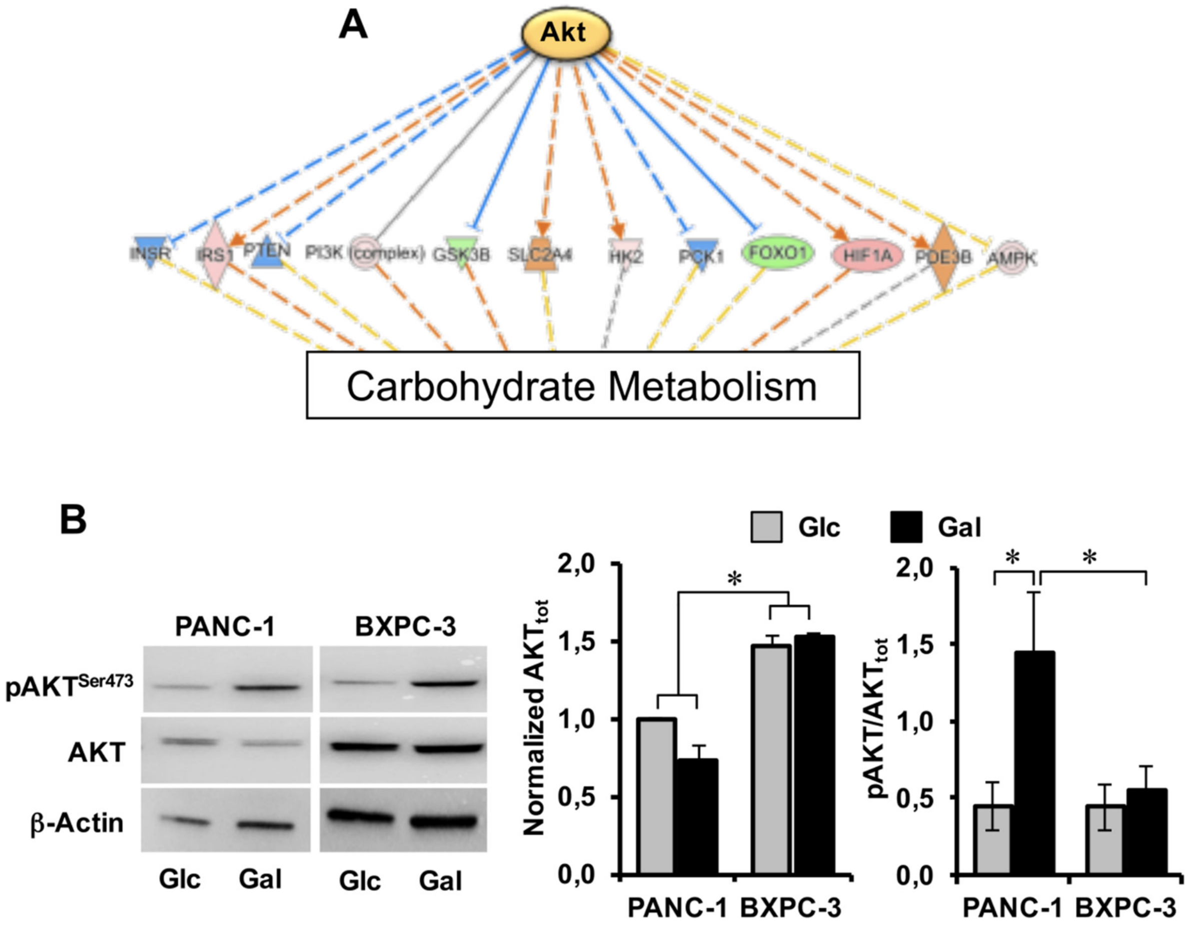

Supplementary Figure 1: *In silico* regulator analysis in PANC-1 and BXPC-3 cell lines. (A) *In silico* regulator effect analysis of differentially expressed genes between BXPC-3 and PANC-1 cells. AKT is predicted as regulator of genes involved in carbohydrate metabolism. Arrow-ending and block-ending lines represent activated and inhibited targets respectively. The analysis was performed using IPA software. **(B)** Protein expression level of AKT and its phosphorylation state. Left panel: representative immunoblot of total cell protein extract from PANC-1 and BXPC-3 with anti-pAKTSer473 (upper lanes), anti-AKT (middle lanes) and anti-β-actin (lower lanes) as protein loading normalizer. Glc, cells grown in complete medium with 11.1 mM glucose; Gal, cells grown for 24 h in medium with 11.1 mM galactose substituting glucose. Histograms on the right: densitometric analysis of total AKT normalized to β-actin and of the pAKT/AKT ratio; the values are means ± SEM of 3 biological replicates under each condition; *, $P < 0.01$.

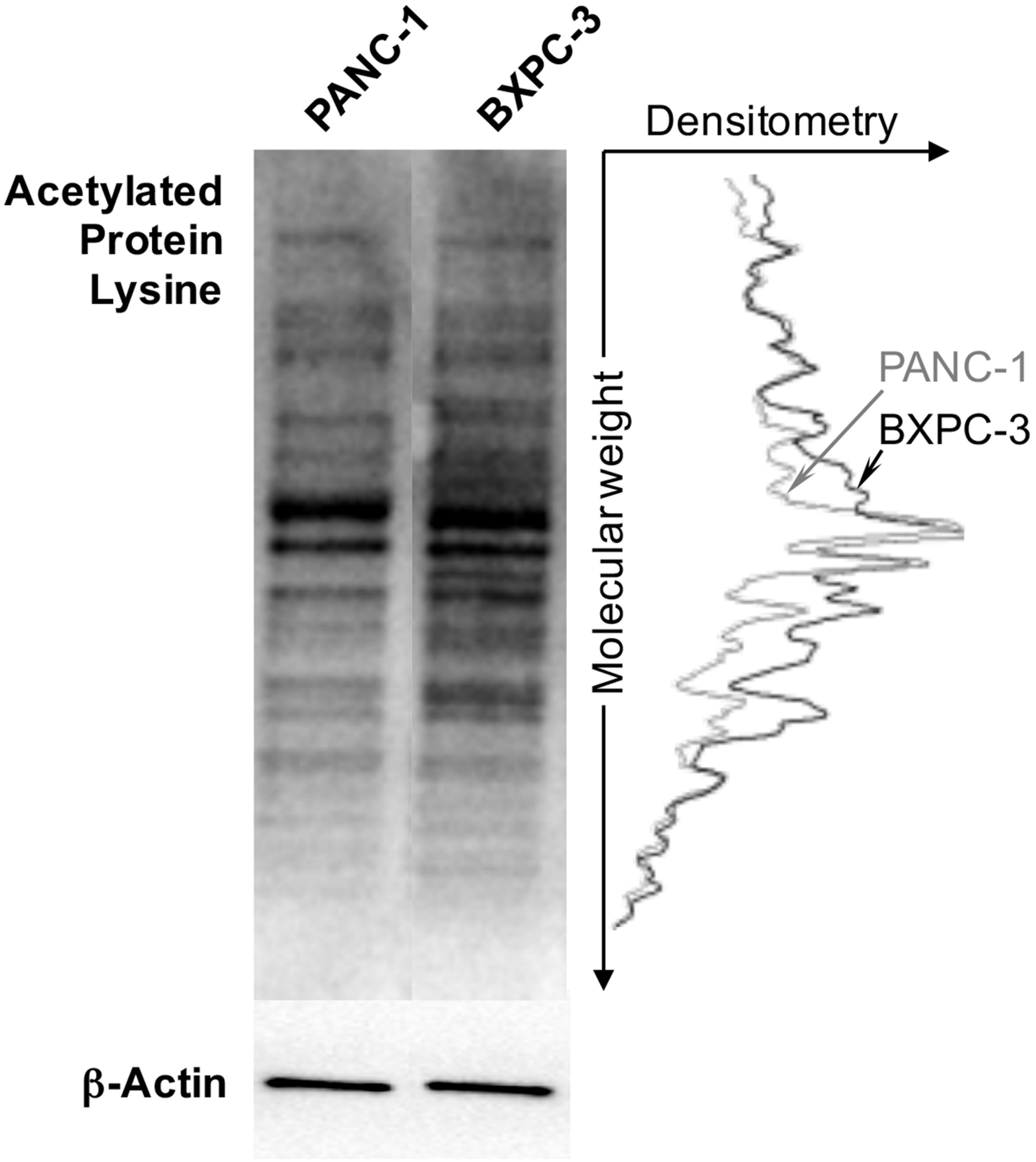

Supplementary Figure 2: Western blotting analysis of acetylated protein lysines in PANC-1 and BXPC-3 pancreatic acncer cell lines. Total cell protein extracts were subjected to western blotting as detailed under Materials and Methods in the main text. The antibodies used were for Acetylated Lysine and β-actin as loading control. The shown western blottings of PANC-1 and BXPC-3 cell proteins along with the relative densitometric profiles is representative of 3 independent biological replicates yielding comparable results.

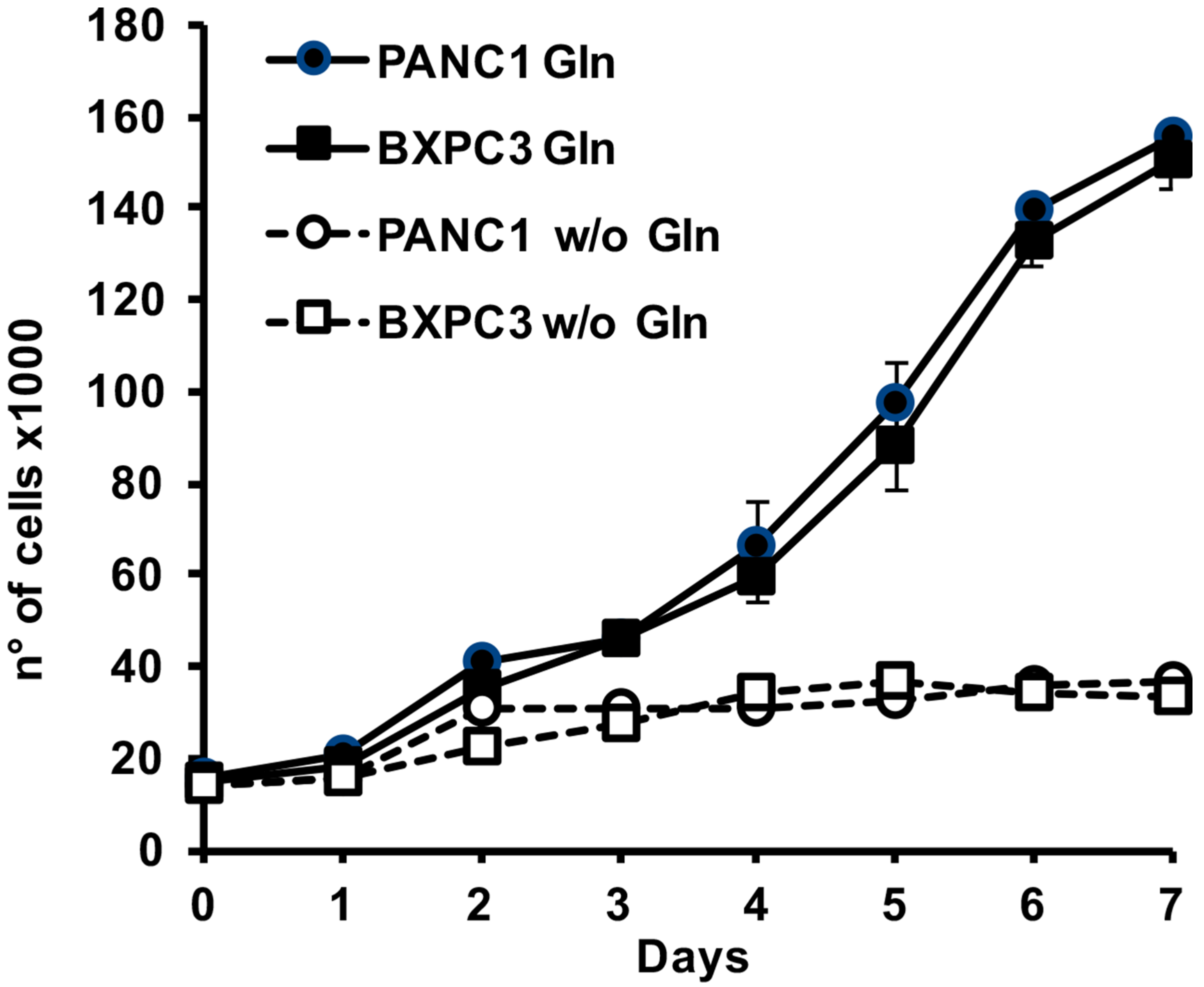

Supplementary Figure 3: Effect of glutamine on the cell growth of PANC-1 and BXPC-3 pancreatic cancer cell lines. Both cell lines were seeded at the same density in RPMI medium containing 11.1 mM glucose with or without 2 mM glutamine; cells were counted every 24 h at the indicated times and the values shown are means of three independent time-courses under each condition.

Supplementary Table 1: List of up-regulated and down-regulated genes in BXPC-3 as compared with PANC-1. Differently expressed genes were selected with differential score (DiffScore) cutoff set at ±13 ($p<0.05$) and logFC of +/- 0.5

See Supplementary File 1
